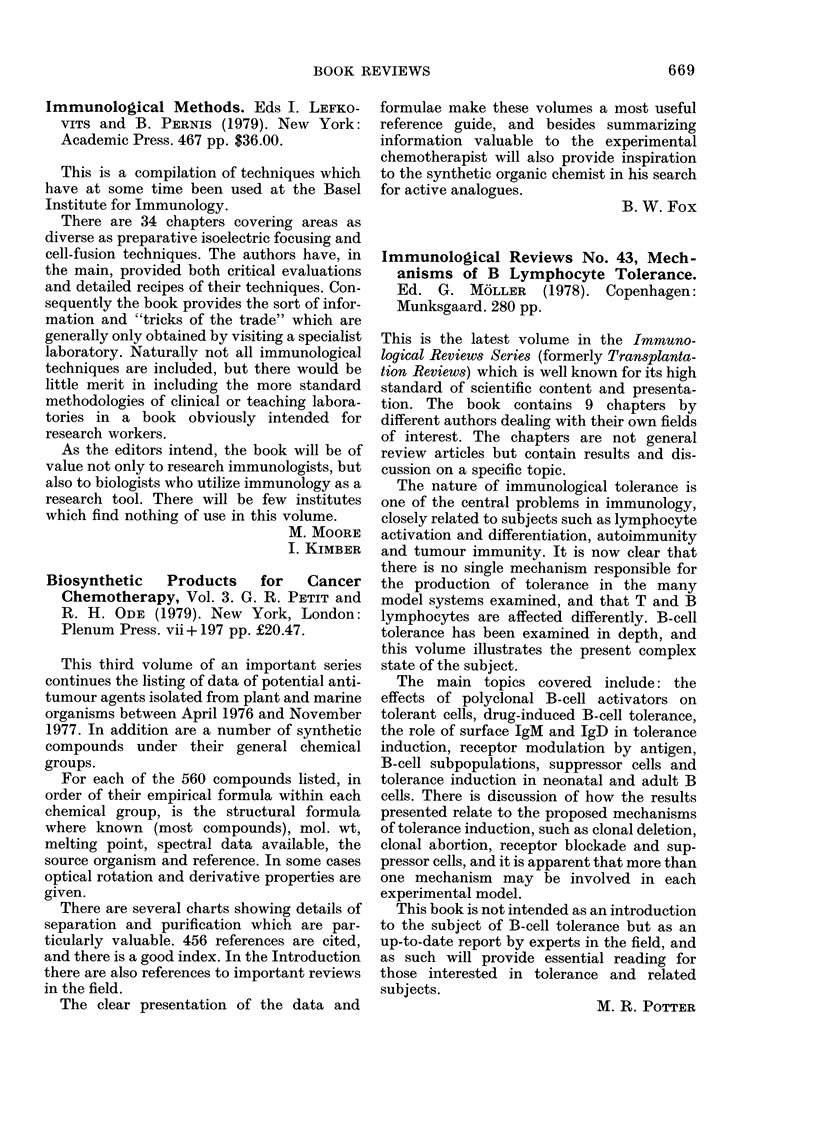# Immunological Reviews No. 43, Mechanisms of B Lymphocyte Tolerance

**Published:** 1979-10

**Authors:** M. R. Potter


**Immunological Reviews No. 43, Mechanisms of B Lymphocyte Tolerance.** Ed. G. MÖLLER (1978). Copenhagen: Munksgaard. 280 pp.

This is the latest volume in the *Immunological Reviews Series* (formerly *Transplantation Reviews*) which is well known for its high standard of scientific content and presentation. The book contains 9 chapters by different authors dealing with their own fields of interest. The chapters are not general review articles but contain results and discussion on a specific topic.

The nature of immunological tolerance is one of the central problems in immunology, closely related to subjects such as lymphocyte activation and differentiation, autoimmunity and tumour immunity. It is now clear that there is no single mechanism responsible for the production of tolerance in the many model systems examined, and that T and B lymphocytes are affected differently. B-cell tolerance has been examined in depth, and this volume illustrates the present complex state of the subject.

The main topics covered include: the effects of polyclonal B-cell activators on tolerant cells, drug-induced B-cell tolerance, the role of surface IgM and IgD in tolerance induction, receptor modulation by antigen, B-cell subpopulations, suppressor cells and tolerance induction in neonatal and adult B cells. There is discussion of how the results presented relate to the proposed mechanisms of tolerance induction, such as clonal deletion, clonal abortion, receptor blockade and suppressor cells, and it is apparent that more than one mechanism may be involved in each experimental model.

This book is not intended as an introduction to the subject of B-cell tolerance but as an up-to-date report by experts in the field, and as such will provide essential reading for those interested in tolerance and related subjects.

M. R. POTTER